# Association of bevacizumab and stroke in ovarian cancer: a systematic review and meta-analysis

**DOI:** 10.3389/fnins.2023.1187957

**Published:** 2023-06-09

**Authors:** Li Song, Yan Liu, Zhixin Chen, Zeyan Li, Shiqin Zhu, Yingjie Zhao, Huihui Li

**Affiliations:** ^1^Department of Obstetrics and Gynecology, Qilu Hospital of Shandong University, Jinan, Shandong, China; ^2^Department of Rheumatology and Clinical Immunology, Juxian People's Hospital, Rizhao, Shandong, China; ^3^Cheeloo College of Medicine, Shandong University, Jinan, Shandong, China; ^4^Department of Rheumatology, Qilu Hospital of Shandong University, Jinan, Shandong, China; ^5^Shandong Provincial Clinical Research Center for Immune Diseases and Gout, Jinan, Shandong, China

**Keywords:** ovarian cancer, bevacizumab, stroke, meta-analysis, zero outcome events

## Abstract

**Background:**

The prognosis for patients with ovarian cancer is bleak. Clinical trials have shown the efficacy of bevacizumab in ovarian cancer treatment. However, life-threatening strokes may limit the usage of bevacizumab and require specific follow-up strategies. This study aims to systematically evaluate the risk of stroke of bevacizumab treatment in ovarian cancer.

**Methods:**

We retrieved all relevant articles published up to December 4th, 2022, from Embase, PubMed, Web of Science, and the Cochrane Library. The risk of stroke in patients with ovarian cancer treated with bevacizumab combined with chemotherapy was analyzed. Meta-analysis was performed using the Stata 17 software and R 4.2.1 program.

**Results:**

Six randomized controlled trials (RCTs) of bevacizumab combined with chemotherapy or chemotherapy for ovarian cancer and six single-experimental-arm trials were included in this study. The meta-analysis showed a pooled risk ratio (RR) of 2.14 [95% confidence interval (CI): 0.88–7.99] for patients with ovarian cancer treated with bevacizumab combined with chemotherapy. Subgroup analyses showed that the incidence of stroke-related adverse events in the carboplatin + paclitaxel + bevacizumab group was 0.01% (95% CI: 0.00–0.01, *p* < 0.01). The incidence of stroke-related adverse events was 0.01% (95% CI: 0.00–0.01, *p* < 0.01) in patients aged ≥60. The incidence of stroke caused by cerebral ischemia and cerebral hemorrhage was 0.01% (95% CI: 0.01–0.02, *p* = 0.27) and 0.01% (95% CI: 0.00–0.01, *p* < 0.01), respectively.

**Conclusions:**

This meta-analysis indicates that chemotherapy combined with bevacizumab may not increase the incidence of stroke in patients with ovarian cancer. However, stroke-related adverse events may be higher in older patients. Cerebral hemorrhage might cause the incidence of stroke more than cerebral ischemia.

**Systematic review registration:**

PROSPERO (CRD42022381003).

## 1. Introduction

Ovarian cancer is the eighth most common malignancy in women and the leading cause of mortality among female genital malignancies (Siegel et al., 2018). Worldwide, there are about 22,240 new cases annually, and the risk of ovarian cancer increases with age (Siegel et al., 2018). Because the early symptoms of ovarian cancer are not obvious and there is a lack of effective screening methods, many advanced patients will eventually develop distant metastasis. Remote invasion and metastasis are the leading causes of death in patients with ovarian cancer. Paclitaxel and carboplatin are standard chemotherapy regimens for advanced and recurrent ovarian cancer (Tsibulak et al., 2019). Despite recent advances in treating ovarian cancer, the prognosis is still poor. The occurrence and development of ovarian cancer depend on the supply of neovascularization nutrients. Blocking neovascularization is a new therapeutic strategy to inhibit tumor growth.

In 1971, Folkman proposed the tumor theory of targeted therapy by inhibiting the formation of tumor neovascularization for the first time. Unlike chemotherapy drugs that act directly on tumor cells, anti-angiogenic therapy acts on the tumor microenvironment, which can degrade tumor blood vessels and inhibit neovascularization. Anti-angiogenic drugs combined with chemotherapy or other targeted drugs may have a better anti-tumor effect (Folkman, 1971). Targeted vascular endothelial growth factor (VEGF) or vascular endothelial growth factor receptor (VEGFR) drugs developed based on the mechanism of blocking tumor angiogenesis are collectively referred to as anti-angiogenic drugs, including macromolecular monoclonal antibody drugs and their bioanalogues, competitive receptor drugs, small molecule receptor tyrosine kinase inhibitors, and non-receptor tyrosine kinase inhibitors.

Bevacizumab is the first large molecule monoclonal anti-angiogenic targeting drug of humanized IgG1 monoclonal antibody targeting VEGF, which is effective in many types of cancer and has been used in gynecological tumors (Monk et al., 2016). Gynecologic Oncology Group (GOG) 0218 is the first successful Phase III clinical study of bevacizumab in ovarian cancer (Burger et al., 2011). Based on the results of the GOG0218 study, European Medicines Agency (EMA) approved bevacizumab for the initial diagnosis of advanced ovarian cancer.

Clinical trial data have indicated that bevacizumab can significantly prolong overall survival (OS) and progression-free survival (PFS) in patients with advanced and relapsed ovarian cancer. However, adverse events in cerebrovascular, especially life-threatening stroke, have been reported in randomized controlled trials (RCTs) (Cannistra et al., 2007; Garcia et al., 2008; Konner et al., 2011; Perren et al., 2011; Aghajanian et al., 2012; Eisenhauer et al., 2014; Herzog et al., 2014; Tewari et al., 2019; Walker et al., 2019; Pfisterer et al., 2020; Pignata et al., 2021; Vergote et al., 2022). Stroke is a collection of disorders that include ischemic and hemorrhagic stroke (which includes parenchymal hemorrhage, ventricular hemorrhage, and subarachnoid hemorrhage) in which a blood vessel in the brain suddenly bursts or limits blood flow to the brain, causing brain tissue damage. The abnormality of tumor blood vessels causes that the surrounding tumor tissue is prone to structural abnormalities during bevacizumab usage, which leads to the loss of support of the vascular structure, resulting in bleeding. This includes the intracerebral hemorrhage that we investigated. Furthermore, bevacizumab can inhibit VEGF and promote platelet accumulation, leading to thromboembolism. Clinically, the most common arterial thrombotic events are cardiovascular and cerebrovascular events. Our analysis included all types of stroke, assessing dose dependence and determining whether a high-dose bevacizumab regimen posed a higher relative risk assessment than the low-dose regimen.

It is unclear how the usage of bevacizumab in ovarian cancer relates to the overall incidence of stroke, and we conducted a meta-analysis of the published RCTs. This study may help to evaluate the cerebrovascular safety of bevacizumab in ovarian cancer and develop follow-up and adverse treatment strategies for patients.

## 2. Methods

### 2.1. Retrieval strategy

Literature searches were conducted in PubMed, Web of Science, Scopus, and Cochrane Library databases up to December 2nd, 2022. Keywords included stroke, bevacizumab, ovarian neoplasm, ovarian neoplasms, ovarian carcinoma, ovarian carcinomas, and ovarian cancer.

### 2.2. Inclusion criteria and exclusion criteria

#### 2.2.1. Inclusion criteria

The inclusion criteria were as follows: (1) studies evaluating the events of stroke-related such as ischemia, cerebral hemorrhage, and subdural bleeding in ovarian cancer patients treated with bevacizumab; (2) RCTs, controlled clinical trials or prospective trials; (3) studies reported the number of the adverse events; (4) human studies; (5) studies published in English.

#### 2.2.2. Exclusion criteria

The exclusion criteria were as follows: (1) abstracts, reviews, animal studies, meta-analyses, and case reports; (2) studies that did not report the selected outcomes or studies that reported the total number of events. Meta-analysis has been registered with PROSPERO (CRD42022381003).

### 2.3. Data extraction and quality assessments

All articles were included, and data were extracted independently by two investigators. The following information was recorded for each study: first author's name, year of publication, trial phase, tumor classification or staging, number of patients enrolled, treatment arms, control arms, median age, median following-up time, number of patients available for analysis, number of events of the stroke-related adverse events.

### 2.4. Statistical analysis

The meta-analysis was performed on eligible studies by calculating the cumulative incidence and 95% confidence interval (CI) for all stroke-related adverse events. Due to zero events in the included study, we performed an arcsine conversion of the original rate before analysis. For the control group, we compared the proportion of patients with stroke-related adverse events who received bevacizumab with the proportion in the control group. The data were expressed as hazard ratio (RR) and 95% CI for dichotomous outcomes. In the analysis, we used a random-effect model or fixed-effect model to perform subgroup analyses for different adverse events, treatment regiments, treatment lines, and ages. Heterogeneity between the studies was assessed by *I*^2^ statistics, with a threshold *p* < 0.05. Homogeneous data (*I*^2^ < 50%) were summarized and analyzed by the fixed-effect model, while heterogeneous data (*I*^2^ ≥ 50%) were summarized and analyzed by the random-effect model. Meta-analysis was performed using the Stata software (StataCorp LLC, College Station, TX; version 17) and R 4.2.1 (Cochrane, London, UK).

The funnel plot (Egger's test) was used to evaluate publication bias. Ideally, the distribution of studies in the funnel plot is roughly symmetrical, suggesting that studies have no publication bias. If bias was present, publication bias was assessed through Duval and Tweedy's trim and fill procedure, which imputes studies from either side of the plotted graph to identify unbiased effects. The significance level for this study was 5% (*p* < 0.05).

## 3. Results

### 3.1. Literature search and research characteristics

**Figure 1:**
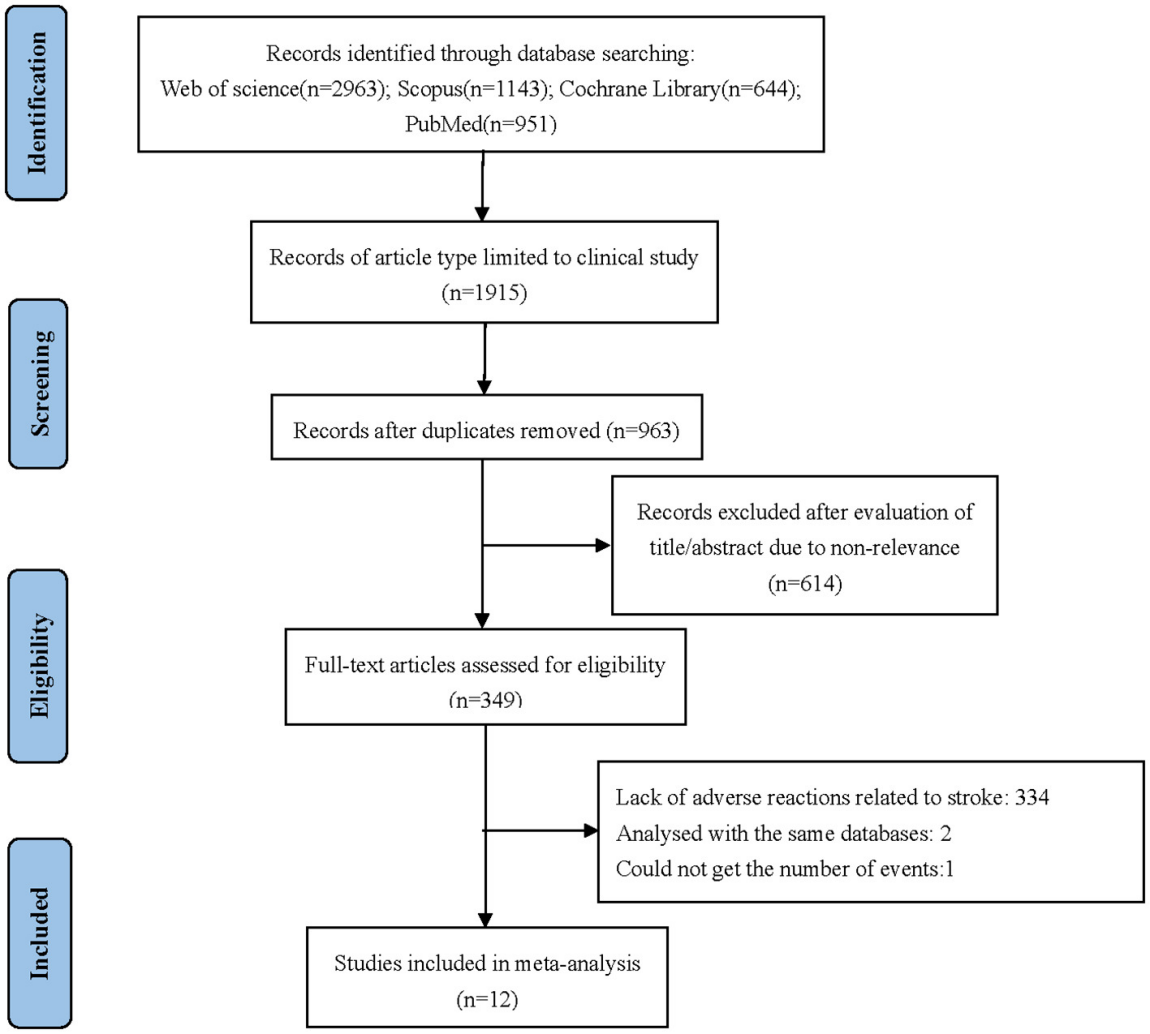
Flow diagram of the study selection process for this systematic review and meta-analysis.

Twelve studies were included in this meta-analysis (Cannistra et al., 2007; Garcia et al., 2008; Konner et al., 2011; Perren et al., 2011; Aghajanian et al., 2012; Eisenhauer et al., 2014; Herzog et al., 2014; Tewari et al., 2019; Walker et al., 2019; Pfisterer et al., 2020; Pignata et al., 2021; Vergote et al., 2022). The specific screening flow chart is shown in Figure 1. From 2007 to 2021, 5,003 patients with ovarian cancer were enrolled, including 3,733 on primary treatment and 1,270 with recurrent ovarian cancer. These clinical studies include six single-experimental-arm studies (Cannistra et al., 2007; Garcia et al., 2008; Konner et al., 2011; Eisenhauer et al., 2014; Herzog et al., 2014; Vergote et al., 2022). In the experimental arm, the treatment regimen is platinum-based chemotherapy combined with bevacizumab. Treatment in the control arm consists of platinum-based chemotherapy combined with placebo. Table 1 summarizes the features of the included studies.

**Table 1:** Baseline characteristics of 12 trials included in the meta-analysis.

**References**	**Study type**	**Cancer type**	**Treatment arm**	**Control arm**	**No. patients**	**Median age, years**	**Mean follow-up, months**	**No. events/total**	
								**Treatment arm**	**Control arm**
Aghajanian et al. (2012)	RCT III	PSROC	Gemcitabine + carboplatin + bevacizumab	Gemcitabine + carboplatin + placebo	480	60	58.2	2/247	2/233
Perren et al. (2011)	RCT III	OC	Paclitaxel + carboplatin + bevacizumab	Paclitaxel + carboplatin	1,498	57	19.4	2/745	0/753
Pfisterer et al. (2020)	RCT III	ROC	Carboplatin–pegylated liposomal doxorubicin + bevacizumab	Gemcitabine + carboplatin + bevacizumab	661	62	12.4	4/332	3/329
Tewari et al. (2019)	RCT III	Stage III–IV OC	Paclitaxel + carboplatin + bevacizumab throughout	Paclitaxel + carboplatin	1,816	60	102.9	2/608	0/601
			Paclitaxel + carboplatin + bevacizumab initiation					0/607	
Eisenhauer et al. (2014)	Single arm II	PSROC	Gemcitabine + carboplatin + bevacizumab	NA	45	60	36	2/45	NA
Konner et al. (2011)	Single arm II	Stage II–III OC	Paclitaxel + carboplatin + bevacizumab	NA	41	53	28.6	1/41	NA
Pignata et al. (2021)	RCT III	PSROC	Carboplatin-based doublet + bevacizumab	Carboplatin-based doublet	406	NA	20.1	1/203	0/203
Vergote et al. (2022)	Single arm III	Stage IV OC	Paclitaxel + carboplatin + bevacizumab	NA	73	76	9.3	1/73	NA
Walker et al. (2019)	RCT III	Stage II–IV OC	Paclitaxel + IV carboplatin + bevacizumab	Paclitaxel + IP cisplatin + bevacizumab	1,529	55	84.8	4/511	10/508
			Paclitaxel + IP carboplatin + bevacizumab					3/510	
Garcia et al. (2008)	Single arm II	ROC	Oral cyclophosphamide + bevacizumab	NA	70	60	23.2	2/70	NA
Cannistra et al. (2007)	Single arm II	PROC	Bevacizumab	NA	44	59.5	10.2	1/44	NA
Herzog et al. (2014)	Single arm II	Stage IB-IV OC	Oxaliplatin + docetaxel + bevacizumab	NA	130	58	47.2	1/130	NA

RCT, random control trial; PSROC, platinum sensitive recurrent ovarian cancer; OC, ovarian cancer; ROC, recurrent ovarian cancer; PROC, platinum resistant ovarian cancer; IV, intravenous; IP, intraperitoneal; NA, not available.

**Figure 2:**
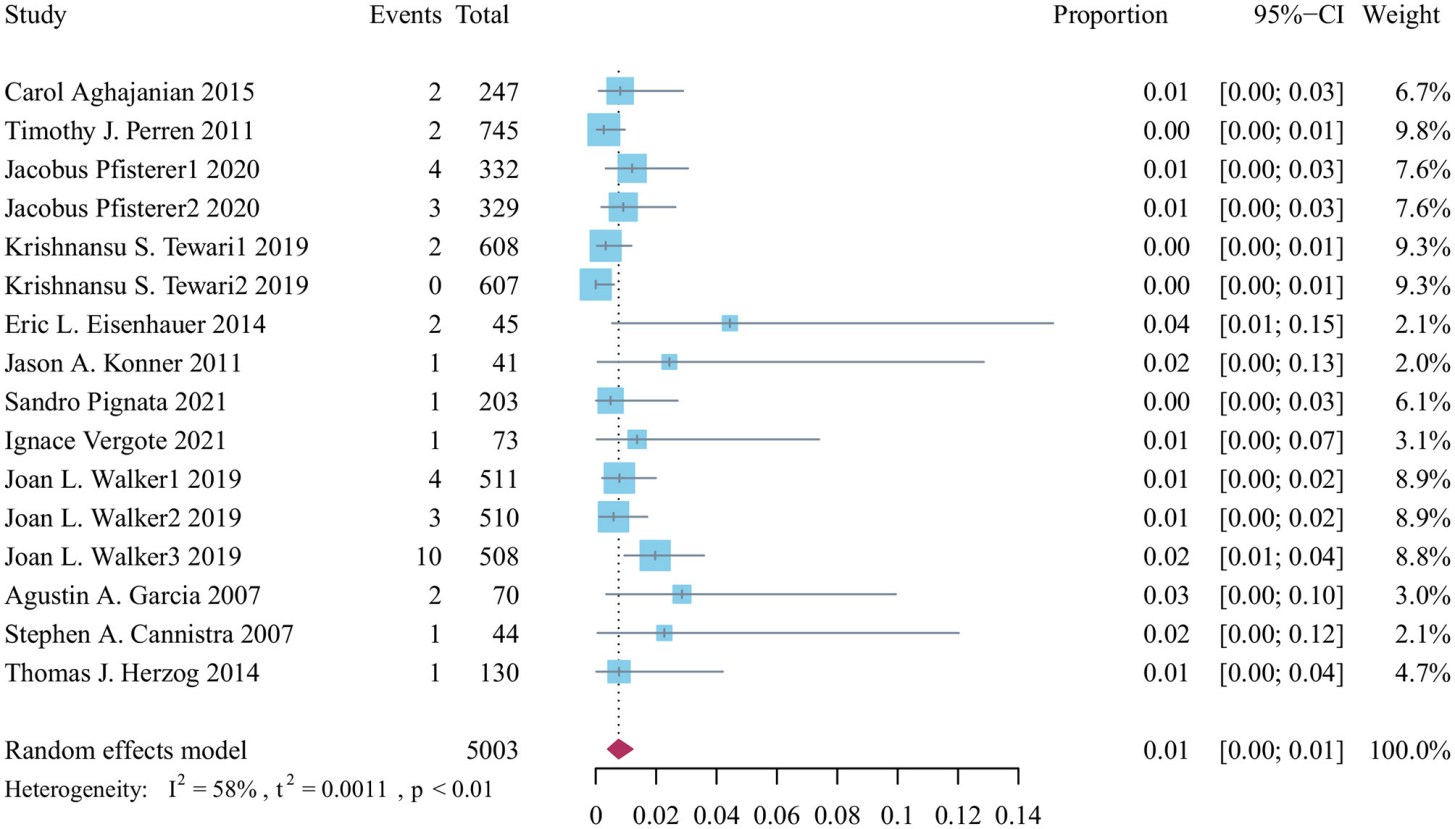
Forest plot of the incidence of stroke related adverse reactions in bevacizumab-treated ovarian cancer patients.

A total of 5,033 patients were treated with bevacizumab, of whom 39 were included in the analysis of stroke-related adverse effects. Based on the results of heterogeneity analysis, the random-effects model was used to calculate the cumulative incidence of stroke-related adverse events to be 0.01% (95% CI: 0.00–0.01, *p* < 0.01; Figure 2).

### 3.2. The risk ratio of stoke-related reactions

**Figure 3:**
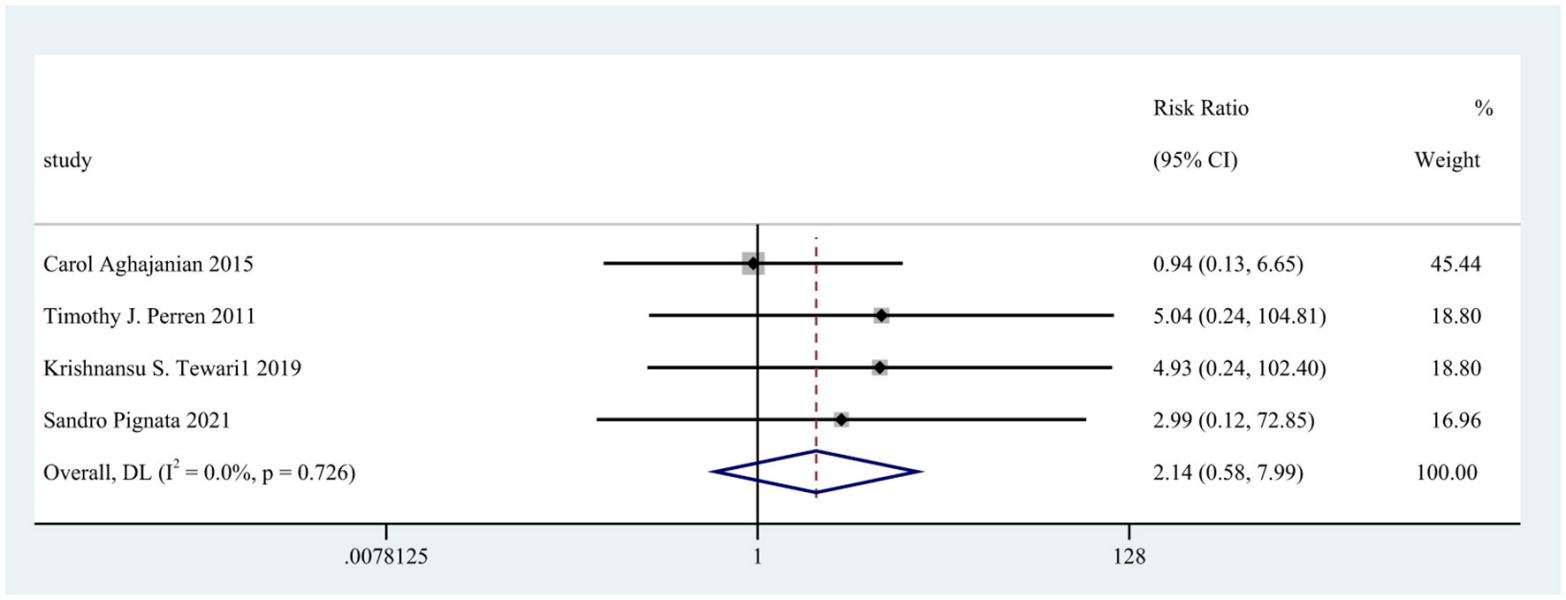
Forest plot of the relative risk of stroke related adverse reactions in bevacizumab-treated ovarian cancer patients.

Four screening studies with five cohorts were controlled trials. Still, the two-arm, zero-event cohort was automatically removed from the data analysis, and four cohorts were included for further analysis of the relative risk of stroke-related adverse effects of bevacizumab in ovarian cancer. The relative risk of 3,593 patients from four cohorts was 2.14 (95% CI: 0.58–7.99, *p* = 0.726; Figure 3).

### 3.3. Publication bias

**Figure 4:**
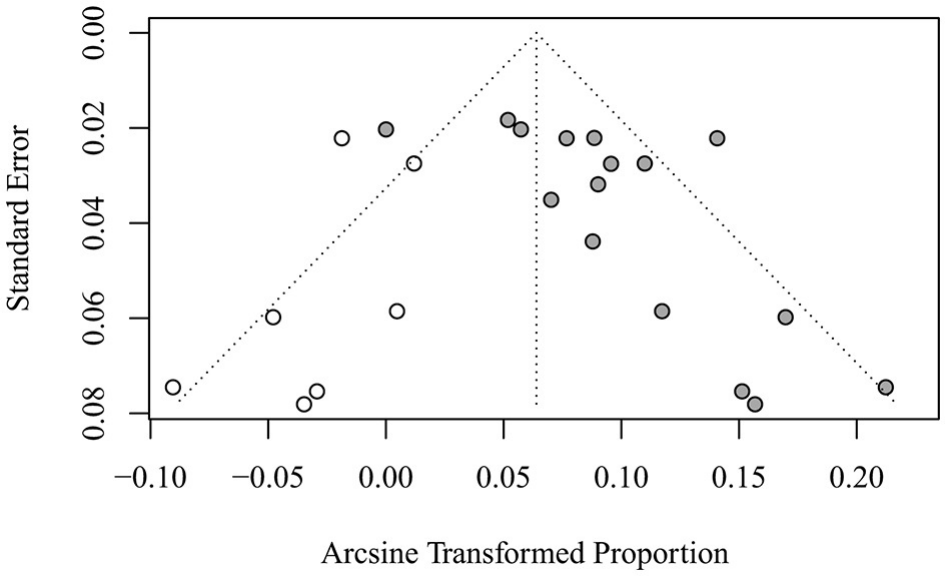
Funnel plot of publication bias of stroke related adverse reactions in bevacizumab-treated ovarian cancer patients.

Publication bias was significant by Egger's test (*p* = 0.03). The trim-and-fill method of Duval and Tweedy was used to identify missing studies based on random-effects models on either side of the average effect in a funnel plot. The results showed that seven studies were missing on the left side of the mean effect (Figure 4) and *p* < 0.0001 after trim and fill. The combined results before and after trim-and-fill were *p* < 0.05, indicating that the results did not change significantly and were stable. The overall random-effects model determined a point estimate with a 95% CI of 0.01 (0.00–0.01) for all combined studies. The trim-and-fill method yielded a point estimate of 0.004 (0.002–0.008).

## 4. Discussion

According to this meta-analysis, bevacizumab in combination with chemotherapy may not make strokes more common in ovarian cancer patients. However, older patients may experience more stroke-related adverse effects. The risk of stroke may be increased more by cerebral hemorrhage than by cerebral ischemia.

Bevacizumab is a recombinant humanized monoclonal antibody against vascular endothelial growth factor (VEGF), which mainly binds with VEGF specifically, then prevents and reduces the binding of VEGF and its receptor, inhibits the activation of VEGF (Monk et al., 2016), thereby inhibiting the formation of tumor new blood vessels, making the blood oxygen supply of tumor tissue insufficient, delaying the growth and metastasis of tumor, and playing an anti-tumor role (Jászai and Schmidt, 2019). In combination with chemotherapy, bevacizumab can also increase vascular permeability, promote the penetration of chemotherapy drugs into tumors, and enhance sensitivity (Zirlik and Duyster, 2018). Published studies have found that overexpression of VEGF can lead to the growth and metastasis of ovarian cancer cells (Orre and Rogers, 1999; Chen et al., 2018). Takeshita et al. (2017) reported that VEGF was related to tumor activity and can be used as a tumor biomarker.

Bevacizumab is an anti-angiogenesis-targeted drug (Monk et al., 2016). An increasing number of clinical trials have demonstrated that bevacizumab is more effective than chemotherapy alone in the first-line or recurrent treatment of ovarian cancer. Based on the results of ICON7 (Perren et al., 2011) and GOG0218 (Burger et al., 2011), the 2019 National Comprehensive Cancer Network (NCCN) guidelines recommend that bevacizumab be added to first-line chemotherapy for ovarian cancer and maintained after completion of chemotherapy. Based on the GOG213 (Coleman et al., 2017) and OCEANS trials (Aghajanian et al., 2012), the addition of bevacizumab to standard chemotherapy and maintenance therapy improved OS and PFS in patients of platinum-sensitive recurrent ovarian cancer. The evidence that ovarian cancer reappears after satisfactory tumor cytoreductive surgery and regular enough chemotherapy to achieve complete clinical remission is called recurrence. In the OCEANS study, gemcitabine plus carboplatin combined with bevacizumab in a single group of patients with platinum-sensitive recurrent ovarian cancer extended the mPFS by 4.0 months (12.4 vs. 8.4 months, HR: 0.48) (Aghajanian et al., 2012). GOG213 studied paclitaxel plus carboplatin combined with bevacizumab, and the results showed that the mPFS was prolonged by 3.4 months, and the median OS was extended by 12.3 months (49.6 vs. 37.3 months, HR: 0.823) (Coleman et al., 2017). The above studies showed that chemotherapy combined with bevacizumab is superior to chemotherapy alone in treating platinum-sensitive ovarian cancer patients. The AURELIA Phase III randomized controlled clinical study on platinum-resistant patients with recurrent ovarian cancer using bevacizumab showed that the median PFS of the combined bevacizumab treatment group was prolonged by 3.3 months (3.4 vs. 6.7 months, HR: 0.42) compared with the paclitaxel weekly treatment/topotecan/PLD monotherapy. The median OS was not significantly prolonged due to cross between groups and other reasons. The subgroup analysis showed that the survival benefit of paclitaxel weekly therapy combined with the bevacizumab group was the most significant (Pujade-Lauraine et al., 2014).

Most RCTs and meta-analyses of bevacizumab have focused on overall or event-free survival. Common toxicities in the description of safety include hypertension, albuminuria, increased risk of thromboembolism, and gastrointestinal toxicity. Rare adverse events such as stroke with bevacizumab were not described. Our study is the first meta-analysis to analyze the incidence of stroke in patients with ovarian cancer. Compared with chemotherapy drugs, bevacizumab does not produce typical cytotoxic effects, and its combination with chemotherapy does not increase chemotherapy-related toxicity. Still, adverse reactions related to anti-neovasculogenesis may occur, such as intracranial hemorrhage and ischemic stroke (Hang et al., 2011). In addition, bevacizumab can damage the integrity of vascular endothelial cells, inhibit the expression of prostaglandin and nitric oxide (Rassaf et al., 2006), promote platelet aggregation, and thus increase the risk of ischemic cerebrovascular events. In addition, bevacizumab can inhibit endothelial cell proliferation and migration, which may damage vascular integrity, resulting in endothelial dysfunction and bleeding (Ferrara, 2004). Alahmari et al. (2016) reported that the combined bevacizumab group was better than the control group. The risk of arteriovenous thrombosis was significantly increased in patients with rectal cancer (RR = 1.334, 95% CI: 1.191–1.494, *p* < 0. 001), and the risk of arterial thrombosis, including stroke, was significantly increased (RR = 1.627, 95% CI: 1.162–2.279, *p* = 0.005) (Alahmari et al., 2016). Other studies showed that the incidence of arterial thromboembolism in bevacizumab combined with chemotherapy was 3.84 and 1.66%, respectively, which increased the risk of arterial thromboembolism compared with chemotherapy alone (Scappaticci et al., 2007). Our study showed that the experimental group's incidence of hemorrhagic and ischemic stroke was higher than the control (*p* > 0.05). Although there was no statistical difference, it suggested that chemotherapy combined with bevacizumab may increase the incidence of hemorrhagic and ischemic stroke.

When evaluating the efficacy of interventions, conventional meta-analysis techniques based on large-sample asymptotic theory can provide good statistical performance. However, due to low event rates and small sample sizes, few or no outcome events were noticed for the evaluation of safety, which led to a zero event (Bhaumik et al., 2012; Efthimiou, 2018). Researchers typically classify zero events into two types: situations in which one or two intervention arms in a single study have zero events are referred to as one-arm zero or two-arm zero events, respectively (Xu et al., 2021a). The most commonly used method to deal with one-arm zero-event studies is continuity correction or its modification (such as empirical correction and reciprocal correction of control samples) (Sweeting et al., 2004), followed by the Peto odds ratio method. And statisticians agree that such studies should not be excluded (Whitehead and Whitehead, 1991; Efthimiou, 2018). However, it is still being determined whether the two-arm zero-event study should be included in the meta-analysis (Böhning et al., 2015). Current studies showed that most of the systematic reviews (98.71%) combined the evidence of one-arm zero-event studies using meta-analysis. However, for two-arm zero-event studies, most (76.23%) were directly excluded from the meta-analysis (Xu et al., 2021b). Some experts believe that whether there is information in meta-analysis in zero-event studies depends largely on the merging methods and assumptions used (Xu et al., 2021b). Therefore, different combined methods should be considered for sensitivity analysis in future meta-analysis. In this study, we incorporate zero-event studies. The Stata software is used to deal with two-arm zero events, and the two-arm zero events are automatically removed during processing. The 'meta' package of R software is used to process one-arm zero events, in which themetaprop function is used to merge rates. Before data analysis, we performed arcsine conversion of the original rate according to the results of normality test (Warton and Hui, 2011) and added 0.5 continuity correction for the study with zero events (Chen and Peace, 2013). Four cohorts were included for further analysis of the relative risk of stroke-related adverse effects of bevacizumab in ovarian cancer. The relative risk of 3,593 patients from four cohorts was 2.14 (95% CI: 0.88–7.99, *p* = 0.726).

Pharmacological toxicity caused by bevacizumab can directly affect patients' tolerance to treatment and thus affect clinical efficacy. Therefore, further studies on the risk factors and mechanisms of bevacizumab-induced stroke are needed. Hypertension is a significant risk factor for stroke. It is necessary to monitor patients' blood pressure dynamically and take antihypertensive drugs regularly. Bevacizumab should be used with caution in patients with a history of arterial thromboembolism, diabetes, or age > 65 years and predisposition to vascular disease (such as a history of heart stenting). Currently, prophylactic use of low molecular weight heparin is controversial and needs to balance benefits and risks (Carrier et al., 2014). Long-term cerebrovascular follow-up is required after treatment with bevacizumab.

There are some limitations in this study. 1. The study's inclusion criteria were strict and included patients with low risk of stroke. 2. It is necessary to conduct a quantitative and comprehensive analysis of the research results of multiple samples based on RCTs to obtain a more credible and consistent conclusion. 3. The non-uniform regime of bevacizumab combined with chemotherapy, such as treatment cycle and combination of different chemotherapy regimens.

## 5. Conclusion

This meta-analysis indicates that chemotherapy combined with bevacizumab may not increase the incidence of stroke in patients with ovarian cancer. However, stroke-related adverse events may be higher in older patients. Cerebral hemorrhage might cause the incidence of stroke more than cerebral ischemia.

## Data availability statement

The original contributions presented in the study are included in the article/Supplementary material, further inquiries can be directed to the corresponding author.

## Author contributions

HL: conception, design, and acquisition of data. LS, ZC, ZL, and SZ: writing and revising the manuscript. YL and YZ: analysis and interpretation of data. All authors contributed to the article and approved the submitted version.
