# Supplementary material for: Association of bevacizumab and stroke in ovarian cancer: a systematic review and meta-analysis
Source: Front Neurosci. 2023 Jun 9;17:1187957. doi: 10.3389/fnins.2023.1187957 (PMC10289163; doi:10.3389/fnins.2023.1187957)
Supplement: Supplementary file 1 [file Data_Sheet_1.PDF]

Supplementary Material

Association of bevacizumab and strokein ovarian cancer: a systematic review and meta-analysis

Li Song[1], Yan Liu[1], Zhixin Chen[2], Zeyan Li[3], Shiqin Zhu[3], Yingjie Zhao[4,5], Huihui Li[1*]

*** Correspondence:** Huihui Li: hhli_qilu@email.sdu.edu.cn

1 Subgroup analysis

We conducted separate subgroup analyses to explore whether different treatment regimens, treatment lines, ages, and adverse reactions were associated with the risk of stroke-related adverse reactions. Among them, the incidence of stroke-related adverse events of bevacizumab was 0.01% (95% CI: 0.00-0.01, $p< 0.01$) in newly diagnosed patients(Figure S1A) and 0.01% (95% CI: 0.01-0.02, p=0.55) in patients with recurrent ovarian cancer (Figure S1B). In addition, subgroup analyses showed that the incidence of stroke-related adverse events in the carboplatin + paclitaxel + bevacizumab group was 0.01% (95% CI: 0.00-0.01, $p< 0.01$; Figure S2). The incidence of stroke-related adverse events was 0.01% (95% CI: 0.00-0.01, p=0.08) in patients aged <60 years (Figure S3A)and 0.01% (95% CI: 0.00-0.01, $p< 0.01$) in patients aged ⩾60 years (Figure S3B). The incidence of stroke caused by cerebral ischemia (Figure S4A)and cerebral bleeding(Figure S4B) was 0.01% (95% CI: 0.01-0.02, p=0.27) and 0.01% (95% CI: 0.00-0.01, $p< 0.01$), respectively.

2 Supplementary Figures

Supplementary Figure 1. Subgroup analysis. (A)Forest plots of the incidence of stroke related adverse reactions in ovarian cancer patients with primary bevacizumab-treat.(B)Forest plots of the incidence of stroke related adverse reactions in ovarian cancer patients in ROC bevacizumab-treat.

Supplementary Figure 2. Subgroup analysis. Forest plot of the incidence of stroke related adverse reactions in paclitaxel + platinum plus bevacizumab-treated ovarian cancer patients.

Supplementary Figure 3. Subgroup analysis.(A)Forest plots of the incidence of stroke related adverse reactions in bevacizumab-treated ovarian cancer patients with age<60. (B) Forest plots of the

incidence of stroke related adverse reactions in bevacizumab-treated ovarian cancer patients with age ⩾60.

Supplementary Figure 4. Subgroup analysis. (A) Forest plots of the incidence of CNS ischemia in bevacizumab-treated ovarian cancer patients.(B) Forest plots of the incidence of CNS bleeding in bevacizumab-treated ovarian cancer patients.
