# Supplementary figures and images for: Association of bevacizumab and stroke in ovarian cancer: a systematic review and meta-analysis
Source: Front Neurosci. 2023 Jun 9;17:1187957. doi: 10.3389/fnins.2023.1187957 (PMC10289163; doi:10.3389/fnins.2023.1187957)

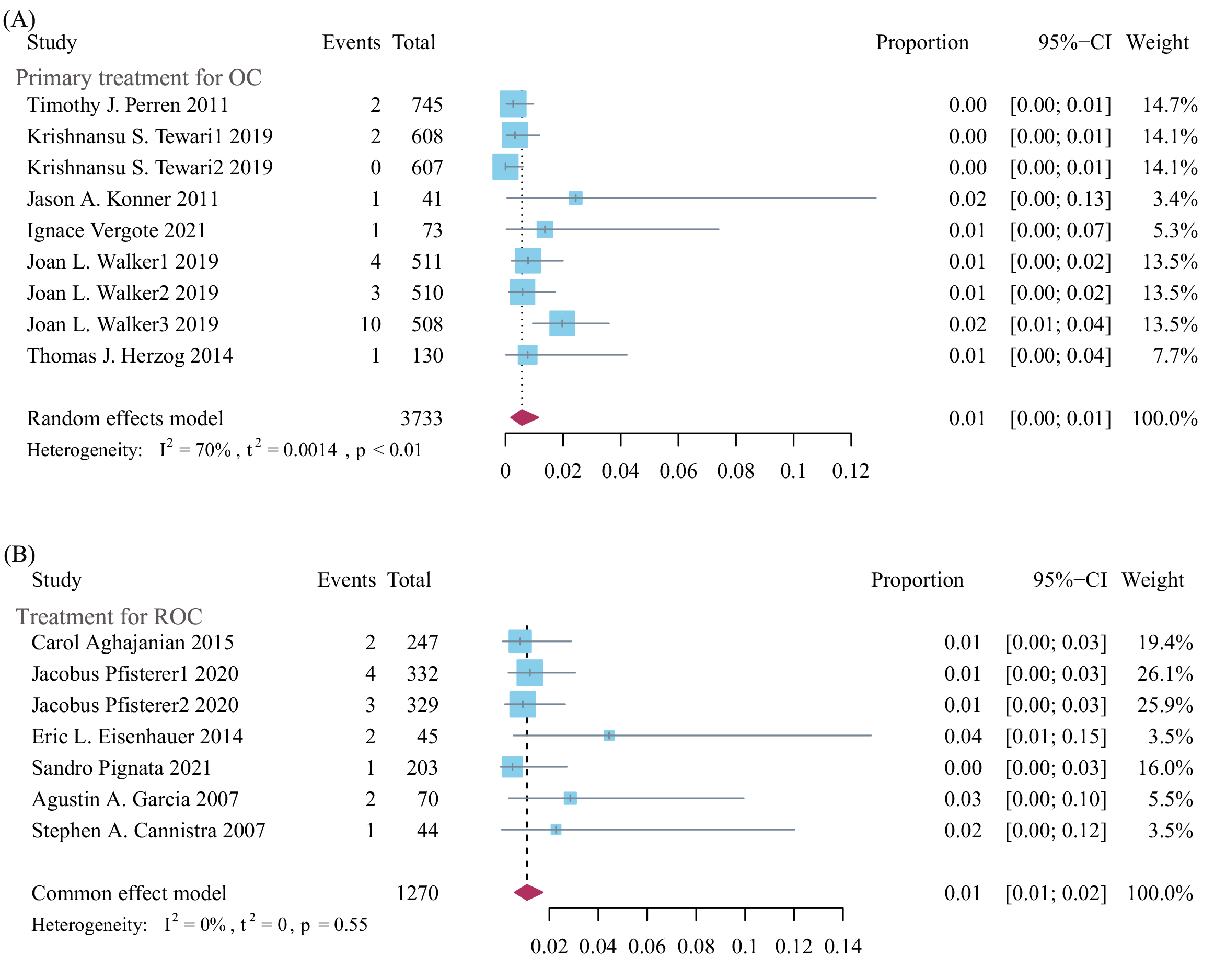

Supplement: Supplementary file 2 [file Image_1.TIF]

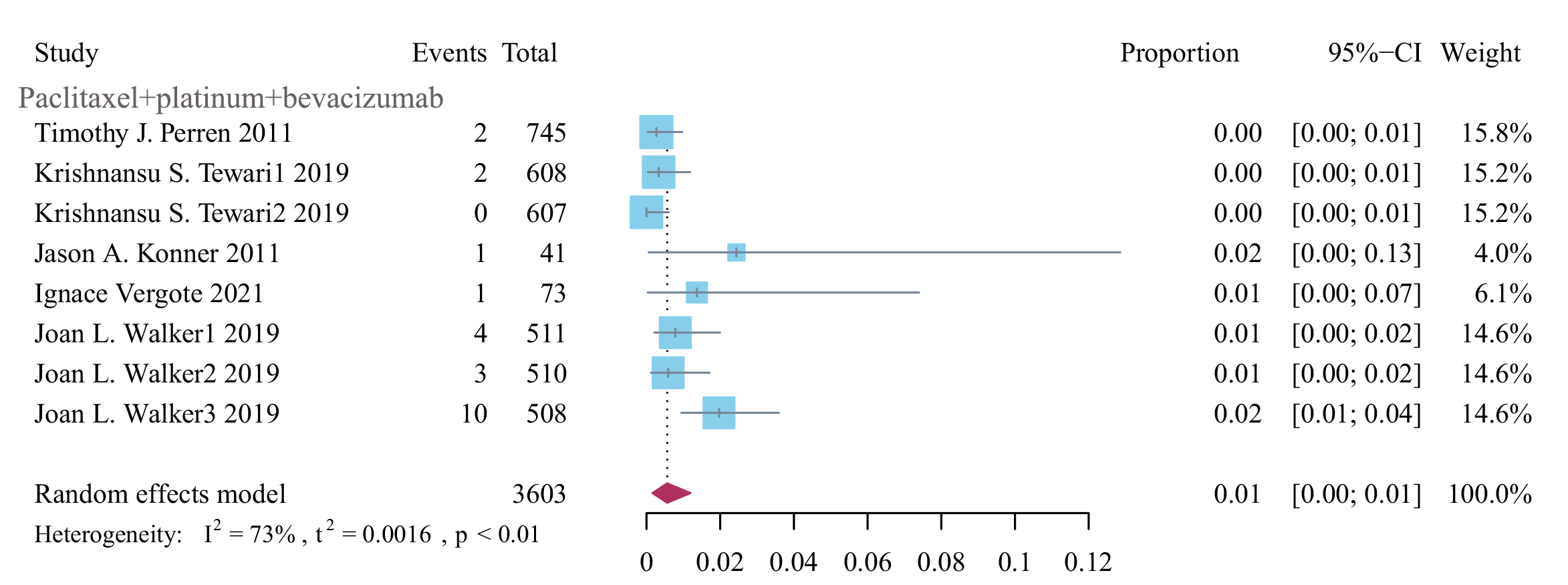

Supplement: Supplementary file 3 [file Image_2.TIF]

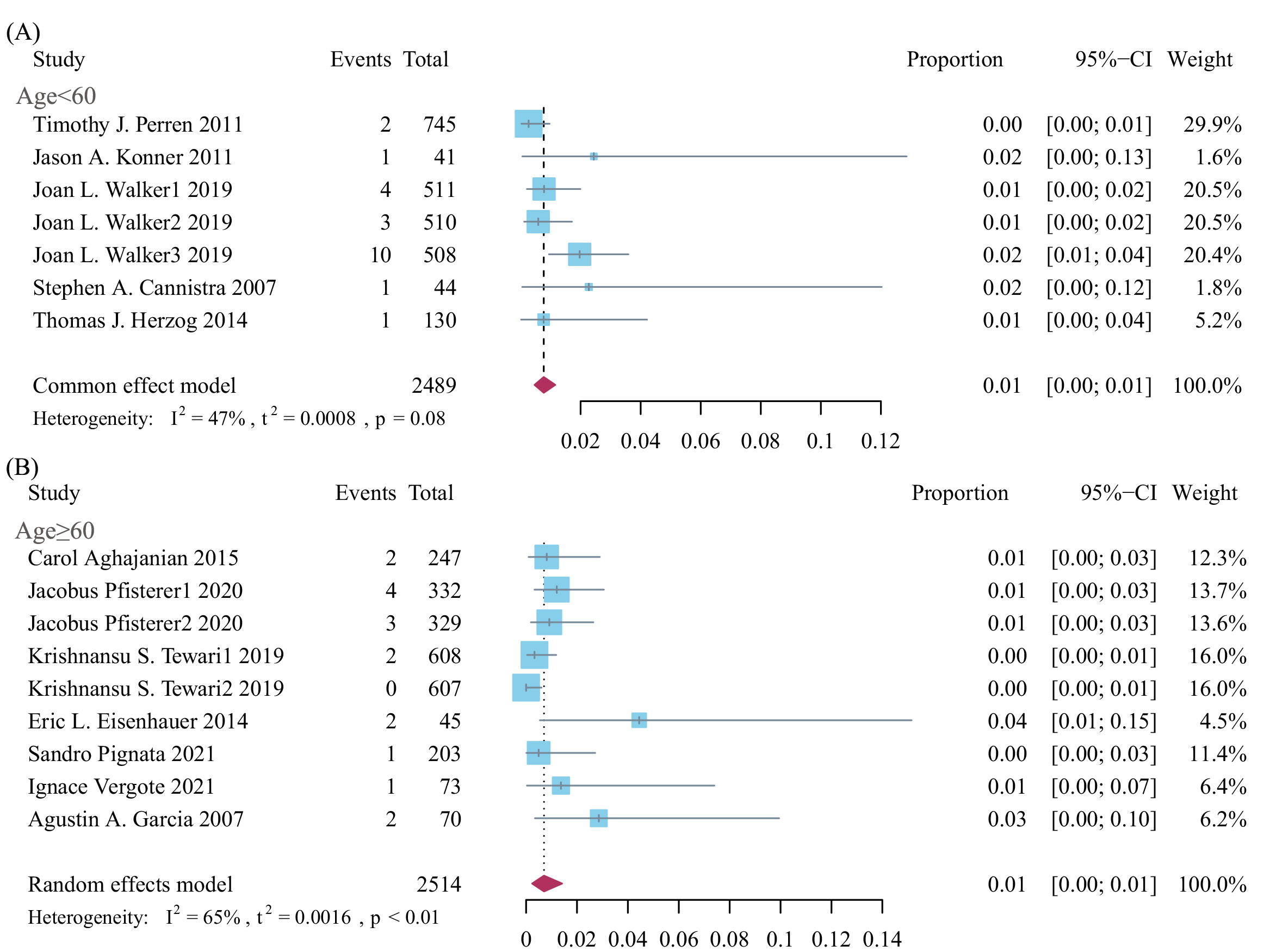

Supplement: Supplementary file 4 [file Image_3.TIF]

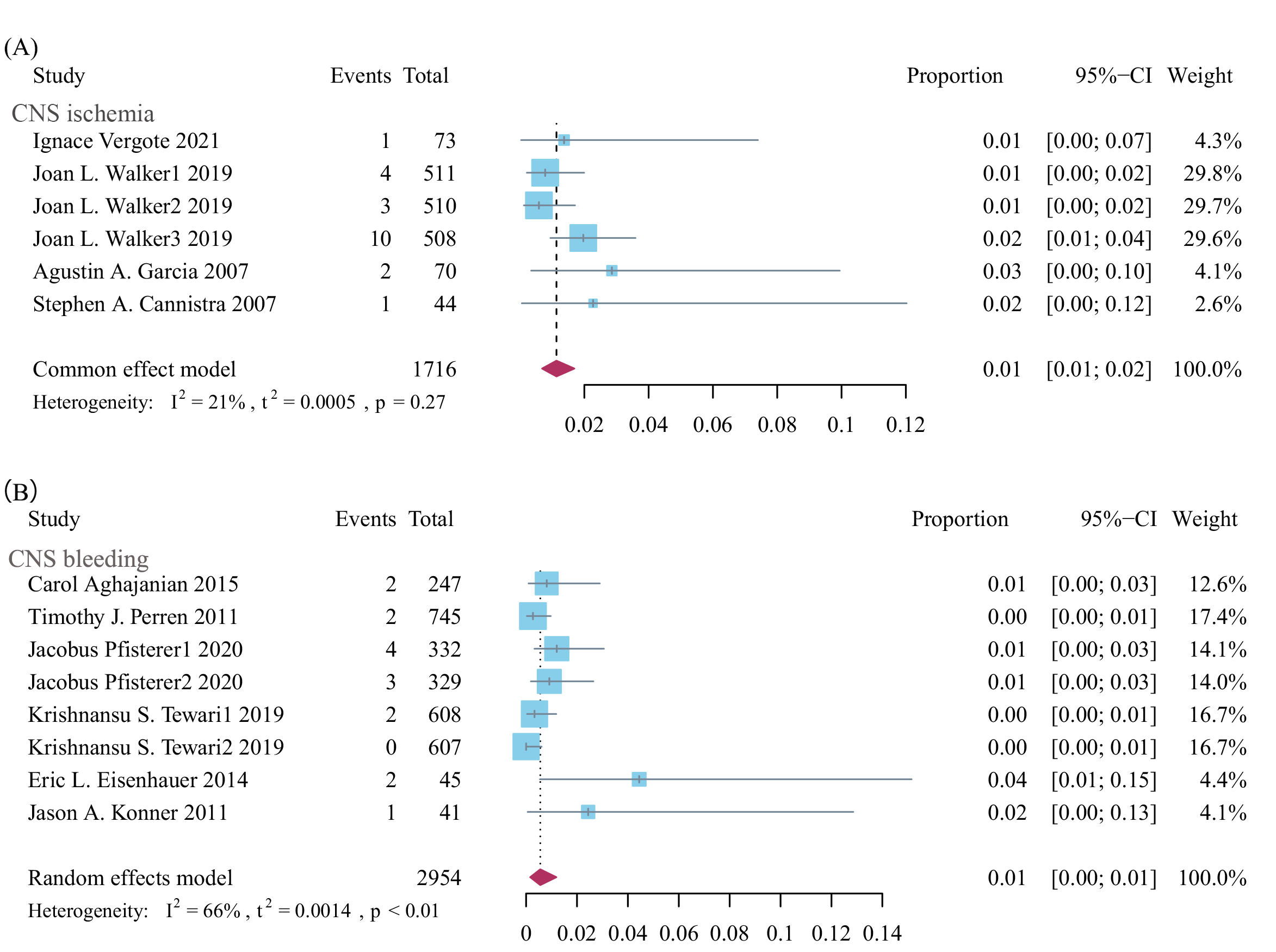

Supplement: Supplementary file 5 [file Image_4.TIF]
